# Dichroic Circular Polarizers Based on Plasmonics for Polarization Imaging Applications

**DOI:** 10.3390/nano11082145

**Published:** 2021-08-23

**Authors:** Junyan Zheng, Xin He, Paul Beckett, Xinjie Sun, Zixin Cai, Wenyi Zhang, Xu Liu, Xiang Hao

**Affiliations:** 1 State Key Laboratory of Modern Optical Instrumentation, College of Optical Science and Technology, Zhejiang University, Hangzhou 310027, China; junyan_zheng@zju.edu.cn (J.Z.); sunxj@zju.edu.cn (X.S.); czx805660580@zju.edu.cn (Z.C.); 21930064@zju.edu.cn (W.Z.); liuxu@zju.edu.cn (X.L.); 2 Intelligent Optics & Photonics Research Center, Jiaxing Research Institute, Zhejiang University, Jiaxing 314000, China; 3 School of Engineering, RMIT University, Melbourne, VIC 3000, Australia; paul.beckett@rmit.edu.au

**Keywords:** metasurface, circular polarizer, polarization imaging

## Abstract

Dichroic circular polarizers (DCP) represent an important group of optical filters that transfer only that part of the incident light with the desired polarization state and absorb the remainder. However, DCPs are usually bulky and exhibit significant optical loss. Moreover, the integration of these kinds of DCP devices can be difficult and costly as different compositions of chemicals are needed to achieve the desired polarization status. Circular polarizers based on metasurfaces require only thin films in the order of hundreds of nanometers but are limited by their sensitivity to angle of incidence. Furthermore, few existing solutions offer broadband operation in the visible range. By using computational simulations, this paper proposes and analyses a plasmonic DCP structure operating in the visible, from 400 nm to 700 nm which overcomes these drawbacks. The resulting circular dichroism transmission (CDT) is more than 0.9, and the maximum transmission efficiency is greater than 78% at visible wavelengths. These CDT characteristics are largely independent of angle of incidence up to angles of 80 degrees.

## 1. Introduction

Unlike amplitude (brightness), wavelength (color), and other light information that the human eye is able to detect without using additional optical devices, polarization information cannot be directly seen. At the same time, circular polarizers are critical optical devices in a broad range of polarized imaging applications such as the classification of biological molecules [1], medical analysis [2], chemical identification [3], quantum information [4], and many other research fields.

Dichroic circular polarizers (DCPs) are an important class of polarizers that attract increasing attention. Their key advantage over other circular polarizers is their ability to absorb the light components with unwanted polarization states instead of reflecting them, thereby reducing interference and other effects on the incident light [5,6]. However, DCPs are usually bulky and can have significant optical loss [6,7,8,9,10]. For example, commercial DCP devices from [9] exhibit transmission efficiencies of around 42% due to the strong absorption of their polymer-based materials. Similarly, while the transmission efficiency of the circular polarizers offered by [10] can reach over 80%, the relative circular dichroism transmission (CDT) is only 0.33 in the visible band. Moreover, this type of circular polarizer needs to use a range of material compositions and/or chemical concentrations to achieve its desired optical properties (e.g., polarization angle and phase difference). Creating polarization sensitive cameras suited to imaging applications [11,12,13] based on these techniques requires advanced overlay methods to integrate the polarizers onto a photodetector array, which is potentially both complicated and costly.

Metasurface-based circular polarizers do not exhibit these drawbacks. Firstly, their component nanostructures are usually fabricated from dielectric or metallic films with thicknesses in the range of a few hundred nanometers and are therefore not bulky [14,15,16,17]. Moreover, the operating wavelengths can be simply tuned by varying the size, spacing, and/or period of the nanostructures [8,15,16,17,18,19,20,21,22,23,24,25,26,27,28,29]. Lastly, metasurface-based optical devices have already been described that have smaller optical loss than existing systems. For example, dielectric and plasmonic metallic nanostructures have been demonstrated in [5,17] with transmission efficiencies of more than 80% and of around 60% in [20,21,30]. As a result, there has been much activity in recent decades exploring metasurface-based polarizers across both academic research and industrial product development. 

Metallic nanogratings are polarization-sensitive nanostructures and, thus, can be used to create linear polarizers [31,32]. Their operating wavelength can be simply tuned by varying the grating period, and the transmission efficiency is directly related to the separation between each nanograting element. The further integration of nanograting-based linear polarizers with different polarization angles [33] onto conventional image sensors has allowed the development of compact linear polarization cameras.

Dichroic linear polarizers (DLPs) based on metallic nanoparticles are also of interest as they behave in the same way as conventional DLPs in that they absorb, rather than reflect, unwanted polarization states [34,35,36,37]. Metallic nanostructures behave as perfect electric conductors with high reflectance, but their optical properties can be simply manipulated. Although much work has been published to demonstrate this basic idea, there have been few demonstrations of metasurface-based circular polarizers working within the visible spectrum. One example is the chiral, two-layer metasurface based on twisted nanorods, described theoretically in [38]. By rotating the second nanorod by 45 degrees, the whole polarizer achieves a maximum transmission difference (ΔT) between the LHP and RHP states of around 58% at 1650 nm. It is also able to distinguish LHP light from RHP light within a broad near IR wavelength range from 1500 nm to 1750 nm by absorbing the RHP component. However, these larger transmission differences can only be achieved at limited wavelengths. 

Stacked multi-layer nanorod array structures with tailored rotational twist were presented in [31] that have operational bandwidths in the visible range from around 500 to 750 nm. However, this structure becomes a phase converter at wavelengths less than 500 nm, swapping the polarization state of the incident light (LCP to RCP or vice versa) by changing its phase difference. Moreover, its constituent gold nanorod material is expensive and is not CMOS-compatible. Circular polarizers based on helical metasurfaces with a high extinction ratio were presented in [39,40,41]. As the metallic structure is asymmetric along the propagation direction, RHP and LHP states can be distinguished over a relatively wide range within and just above the mid-infrared region from around 3 μm to 10 μm [39,40]. However, the types of 3D nanostructures required here are difficult to build using typical nanofabrication facilities such as electron-beam lithography or focused ion beam. An alternative might be a maskless 3-D nano-printing system such as Nanoscribe [42], but in this case the restricted accuracy and feature sizes of these systems (typically >300 nm) will impose limits on the operating range, which is inversely proportional to the size (width, length, etc.) of the nanostructures. 

Double-helical metasurface-based nanostructures have been shown to have a broad operating range across the whole visible spectrum. However, these structures are also very difficult to fabricate using any recent nanofabrication technique [41]. Although direction-controlled bifunctional metasurface Polarizers, formed by inserting nanoslits with two different thicknesses into a thick gold film, have been demonstrated [43] to exhibit wide-band operation from 600 nm to 1000 nm, their thick metal layer results in low transmission efficiency—around 8% in the experiments of [43]. A planar chiral metasurface comprising double-layered dielectric–metal–dielectric resonant structures in the shape of a gammadion has been shown [44] to offer high transmission efficiency, but only over a very narrow band at around 1100 nm. The metasurface polarization camera of [45], based on TiO_2_ nanorods, is capable of simultaneously taking polarized and 3D images with one snapshot. The metasurface can diffract circularly polarized light with four phase differences into four directions. Although the camera is capable of taking full Stokes images, it has two main drawbacks. Firstly, as it operates by diffraction, it will work only at a single wavelength (532 nm), which is determined by the period of the nanostructures. Further, as the four images result from diffracted rays that are therefore not parallel, an aspheric lens is required to realign them. Notwithstanding these issues, this research clearly shows that full Stokes snapshot cameras have potential applications across a number of research fields. In addition to taking 3D images, they can also be used to characterize biological structures with full Stokes images [46]. Various other metasurface-based polarizers have been described, but few of them can operate at visible wavelengths, even after reductions in their period or size [8,24,43,47,48,49,50,51,52,53].

Plasmonic nanostructures were previously introduced as promising candidates for light filtering applications [16,18,19,20,21,22,23,25,26,27,28,29,30,51,54]. Plasmonic behavior emerges in two basic situations [55]. Firstly, surface plasmon polaritons (SPP) occur if the plasmon is excited and propagated at the interface between the metal and dielectric with propagation constant: $\beta =k_{0}\sqrt{\frac{\epsilon_{1}\epsilon_{2}}{\epsilon_{1}+\epsilon_{2}}}$, where $k_{0}=\frac{\omega }{c}=\frac{\frac{2\pi c}{\lambda }}{c}=\frac{2\pi }{\lambda }$, $\epsilon_{1}$ and $\epsilon_{2}$ are the dielectric constants of the dielectric and metal. Secondly, localized surface plasmons (LSP) are evident when the excited plasmons propagate at the surface of the metallic nanoparticle (e.g., nanosphere, nanodisk, etc.), and are still coupled to the electromagnetic field. The behavior of the SPP case will depend largely on the materials chosen, whereas, for the LSP case, the peak wavelength mostly depends on the nanoparticle size [8,24,43,47,48,49,50,51,52,53]. 

Gold (Au), silver (Ag), and aluminum (Al) are the generally preferred materials for plasmonic filters operating in the visible and NIR (VNIS) wavelengths (from 400 nm to 1000 nm) because other metals, such as chromium (Cr), nickel (Ni), tungsten (W), and titanium (Ti), have larger absorption across the VNIS [55]. The resonant wavelength of Au is above 500 nm, preventing its use in filters with target wavelengths less than this value. The strong oxidizing property of Ag also limits its application as its optical properties alter as it oxidizes. In addition, both Au and Ag require a seed layer (e.g., Cr, Ti) to improve their adhesion to silicon and this extra layer serves to reduce the transmission efficiency of the resulting optical filters [14]. Aluminum, as a CMOS compatible material, does not need a seed layer and is therefore more suitable for optical filtering applications that directly interface to a CMOS sensor [16,21,29,30,54].

In this paper, we present our plasmonic DCPs based on a five-layer polarization sensitive nanostructure (Al nanograting and nanocuboid combinations) with the capability to absorb circularly polarized light of an undesired direction. Our plasmonic polarizers use Al and SiO_2_ as the main materials, thus demonstrating good compatibility with CMOS image sensors, making them suitable for polarization imaging applications. Moreover, our DCPs have a wide operating band through the whole visible wavelengths and its CDT is largely insensitive to the angle of incidence between 0° and 80°.

## 2. Design and Simulations

**Figure 1:**
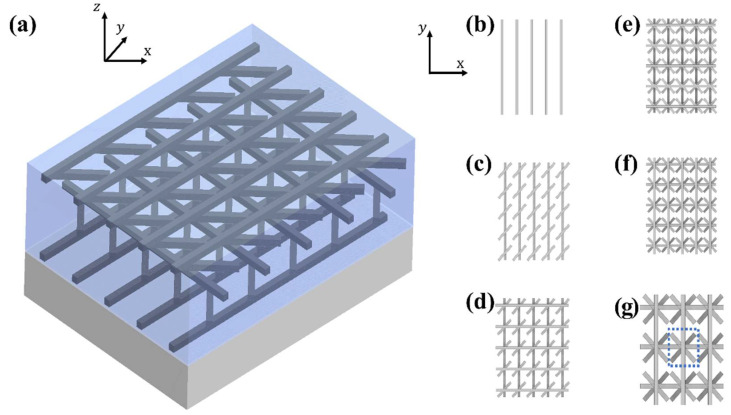
Nanostructures of the dichroic circular polarizer. (**a**) Overall structure in 3D view, showing the relative positions of the layers. Note: each layer is isolated with 30 nm SiO_2_ (**b**) Top layer: Al nanograting aligned along the *y*-axis, (**c**) Second layer: Al nanocuboid array rotated 45° around the *z*-axis, (**d**) Third layer: Al nanograting rotated by 45° so it now aligns with the *x*-axis, (**e**) Fourth layer: Al nanocuboid array, again rotated 45° around *z*-axis, (**f**) Fifth layer: Al nanograting rotated and aligned along the *y*-axis, (**g**) Top view of the dichroic circular polarizer with the simulation unit shown within the blue dashed square.

The overall structure of our dichroic circular polarizer (DCP), which is designed on a quartz substrate, is shown in Figure 1a. The first (top) layer of our DCP in Figure 1b is based on a 30-nm-thick Al nanograting array placed along the y-axis. The grating is 850 nm long and 30 nm wide, and is repeated along the x-direction with a period of 170 nm. Figure 1c shows the second layer made of a 30-nm-thick nanocuboid array, each of them with a width of 30 nm and a length of 170 nm. This layer was designed by rotating each nanocuboid 45° clockwise around the *z*-axis. Aluminum nanogratings and nanocuboids are polarization sensitive, so that incident TE light will be suppressed when its electric field is perpendicular to the repeating nanogratings, or to the longer side of the nanocuboids. The overall metallic nanostructure in Figure 1g is therefore created by continuing to rotate each successive layer by 45° in the same clockwise direction around the *z*-axis, resulting in the structures shown in Figure 1d–f. Note that a 30-nm layer of SiO_2_ was deposited between each metallic nanograting/nanocuboid layer to act as isolation and the whole nanostructure was also encapsulated with SiO_2_.

The simulations were performed using commercial software (COMSOL Multiphysics^®^), applying finite element methods (FEMs). To reduce computation time, the small blue block in Figure 1g was chosen as our simulation unit. Periodic boundary conditions (PBCs) were applied on all four sides and perfectly matched layers (PMLs) were used along the propagation direction, surrounded by scattering boundary conditions to absorb redundant light. Port boundaries were set between PMLs and their adjacent layers, and the LHP and RHP states were excited along the Z direction. The refractive indices of quartz substrate and isolation layers were 1.45 and the refractive index of Al was taken from Rakic’s data [56]. Note that PBCs are replaced with PMLs when simulating a single pixel of the dichroic circular polarizer. 

To compute the results, the transmission efficiency was calculated using the S parameter |S_21_|^2^ (in COMSOL the equation is abs (ewfd.S21^2)), and the CDT (circular dichroism transmission) was determined by the equation: $\mathrm{CDT}=\frac{T_{1}-T_{2}}{T_{1}+T_{2}}$, where *T*_1_ and *T*_2_ are the transmission efficiencies of the two orthogonal polarization states, LHP and RHP, respectively. A higher CDT value means a better capability to detect and distinguish one direction of circularly polarized light from the other.

## 3. Results

**Figure 2:**
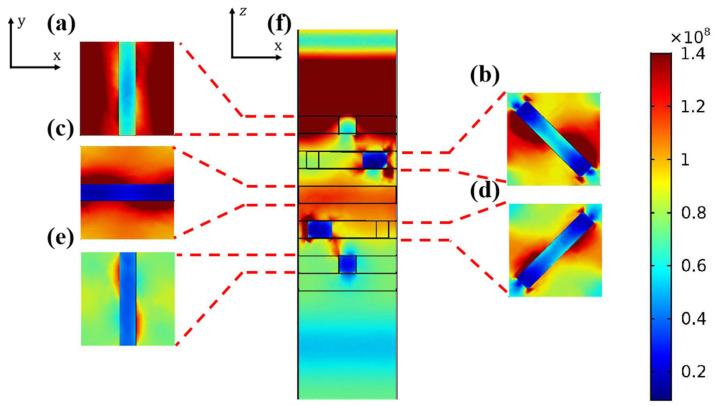
Electric field concentration of our proposed LHDCP for: (**a**) first (top) layer of Al nanograting aligned along the *y*-axis, (**b**) second layer of Al nanocuboids rotated 45° around the *z*-axis, (**c**) third layer of Al nanograting rotated 45° so it now aligns with the *x*-axis, (**d**) fourth layer of Al nanocuboid rotated around the *z*-axis by 45°, (**e**) fifth layer of Al nanograting rotated so it now aligns again with the *y*-axis, (**f**) Electric field cross-section of DCP model in the *x-z* plane.

The electric field shown in Figure 2 indicates the primary working principle for our left-handed DCP (LHDCP). Here, the LHP light is excited from the top. As the Al nanogratings/nanocuboids are polarization-sensitive, it is expected that the electric field will be more concentrated along the two sides in the y-direction than it is in the x-direction. Based on this concept, we built the model of Figure 1a. Figure 2a–e show the resulting electric field concentration seen from the top of the simulation unit of Figure 2f. As expected, most of the electric power is indeed concentrated on the two sides of the Al nanograting/nanocuboid along the diagonal direction. This behavior becomes much clearer as the LHP light propagates further into the structure (Figure 2e).

**Figure 3:**
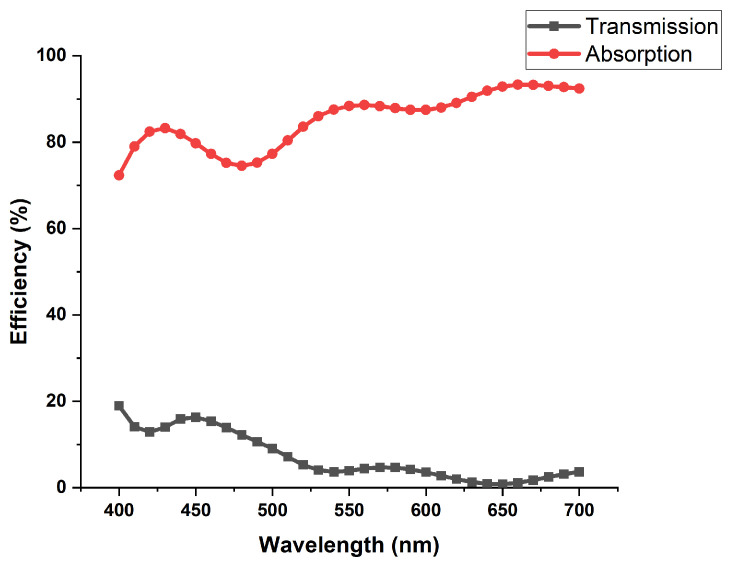
Black and red lines are the transmission and absorption spectra of our LHDCP under RHP incident light.

The transmission and absorption spectra in Figure 3, below, indicate the second working principle of our LHDCP under RHP light. From the graph, it can be seen that instead of reflecting RHP component, our LHDCP absorbs and therefore filters out RHP light. In contrast, the LHP light passes through the filter. As a result, problems such as interference between the incident and reflected light will be virtually eliminated.

**Figure 4:**
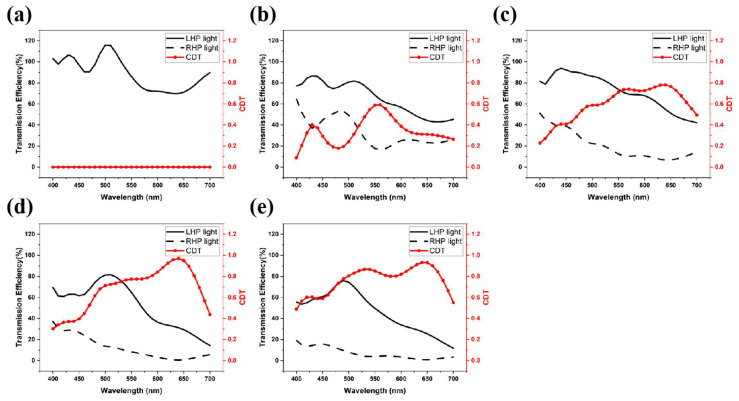
The transmission efficiency of LHP, RHP light and CDT of our proposed nanostructures, the combinations of nanogratings and nanocuboids for: (**a**) 1-layer, (**b**) 2-layer, (**c**) 3-layer, (**d**) 4-layer, (**e**) 5-layer.

As mentioned previously, nanogratings and nanocuboids are sensitive to TM polarized light so that, when the nano-structures are rotated through an angle, the polarization angles of incident TM light change correspondingly. Moving the rotated structures to a different layer will introduce a phase difference. Therefore, LHP and RHP light can be distinguished by adjusting the layer components. Figure 4 describes our investigation between one-layer structure and five-layer structures. Note that the first, third, and fifth layers are nanogratings, whereas the second and fourth are the nanocuboids, previously shown in Figure 1. It can be seen from Figure 4 that, when we increase the number of rotations or layers, the CDT becomes larger and the wavelength range where the CDT greater than 0.5 becomes wider.

**Figure 5:**
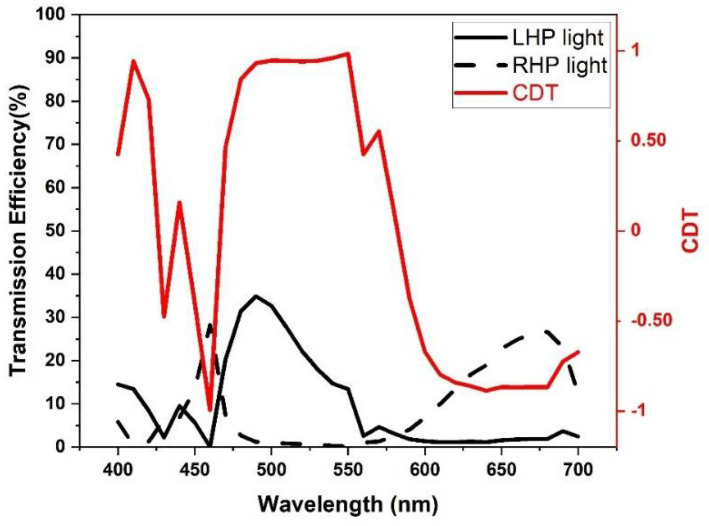
The transmission efficiency of LHP, RHP light and CDT of 5-layer nanocuboids.

The reasoning behind this combination of nanogratings and nanocuboids is as follows. The function of a plasmonic nanograting is based on surface plasmon polaritons (SPP), and it is well known that any metallic nanostructures based on SPP will be sensitive to the angle of incidence. In contrast, the behavior of a nanocuboid surface depends on localized surface polaritons (LSP), which are insensitive to the angle of incidence. As shown in Figure 5, circular polarizers based only on Al nanocuboid arrays cannot achieve a wide operating spectrum across visible wavelengths whereas combinations of nanograting and nanocuboid surfaces are able to both function over a wide bandwidth in the visible and be insensitive to the angle of incidence.

**Figure 6:**
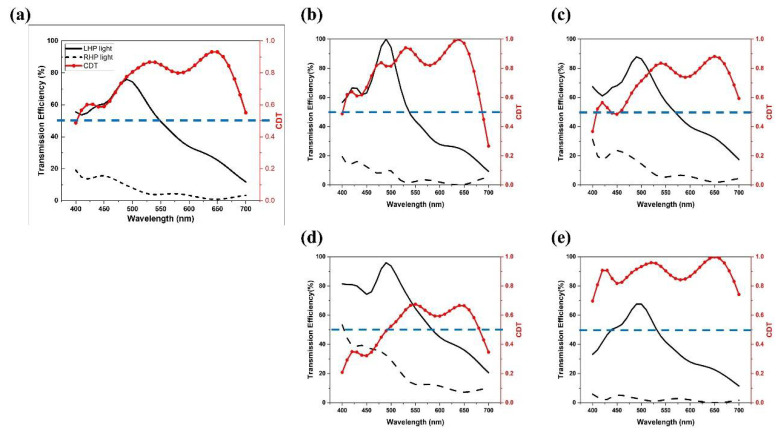
Black lines are the transmission spectra of our LHDCP under LHP and RHP incident light. The red line is the CDT calculated from the above two transmission spectra. Results for: (**a**) Period 170 nm, Width 30 nm, (**b**) Period 160 nm, Width 30 nm, (**c**) Period 180 nm, Width 30 nm, (**d**) Period 170 nm, Width 20 nm, (**e**) Period 170 nm, Width 40 nm.

Based on the principles of the LHDCP outlined above, we optimized the size of our DCPs by varying the width and period parameters of our nanostructures over a narrow range centered on 30 nm and 170 nm, respectively. The final result is shown in Figure 6a and the optimization process is the following: The period was first changed from 170 nm to 160 nm (Figure 6b) and 180 nm (Figure 6c) while maintaining the width at 30 nm. Then, the width was set in turn to 20 nm (Figure 6d) and 40 nm (Figure 6e) while maintaining the period at 170 nm. It can be seen that, as the period increases, the range of wavelengths over which the transmission efficiency of the LHP light is larger than 50% also increases. At the same time, the range where the CDT is larger than 0.5 decreases. On the other hand, increasing the width results in a wider range where the CDT is larger than 0.5, but narrows the transmission efficiency range. Therefore, after evaluating the results in Figure 6a, our optimized width and period parameter were set at their original values of 30 nm and 170 nm, respectively. 

The resulting transmission spectra of the left circular polarizer under LHP and RHP incident light and CDT are shown in Figure 6a. The solid black line is the transmission spectrum of LHDCP when LHP light is incident, which has a maximum transmission efficiency of 78% at 500 nm. The black line is the transmission spectrum of LHDCP under RHP incident light. Although the transmission efficiency decreases when the wavelength is tuned to the red range, the CDT reaches more than 0.9 at 650 nm, and the extinction ratio is 30. The polarizer filters out almost all the RHP light. Therefore, from the CDT results in Figure 6a, our LHDCP is sufficient for polarized imaging applications. The lower transmission efficiency at some wavelengths can be compensated by increasing the exposure time of the image sensor.

**Figure 7:**
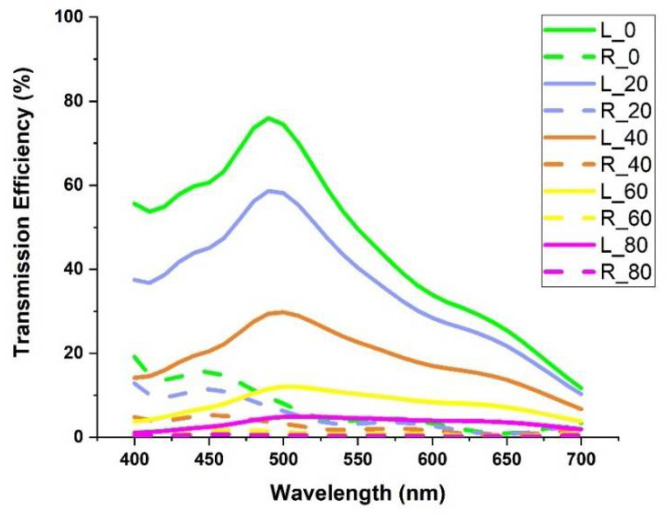
The transmission efficiency of LHDCP with incident LHP and RHP light vs. angle of incidence, varied in 20° steps from 0° to 80°. Solid lines are LHP and dotted lines are RHP.

**Figure 8:**
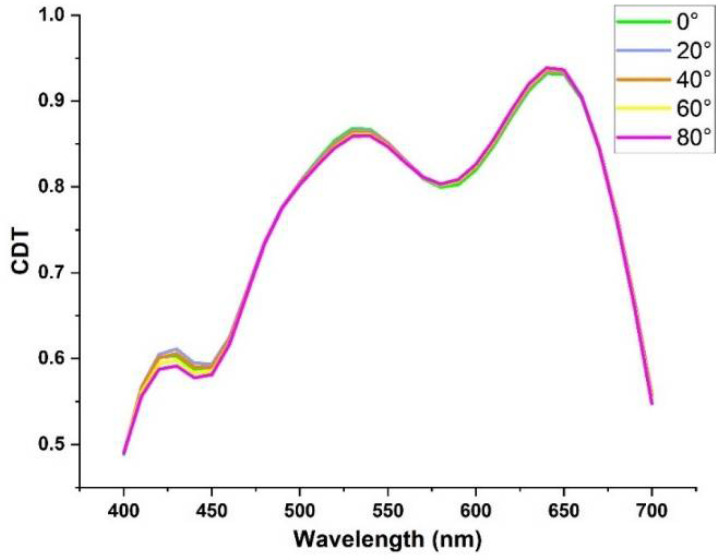
CDT under incident light with different angles from 0° to 80°.

Our LHDCP also exhibits good performance over a wide range of incident angles. We varied the incident angles for both LHP and RHP light show in Figure 7 and calculated the corresponding CDT. Figure 8 shows the CDT lines under incident light with angles up to 80°. As can be seen, the CDT is almost constant with angles over the visible range, indicating that our DCP has a large tolerance to incident angles and will not require a lens with a particular *f*-number to support polarized imaging. Therefore, it is likely to be suitable for a wide range of polarized imaging applications.

**Figure 9:**
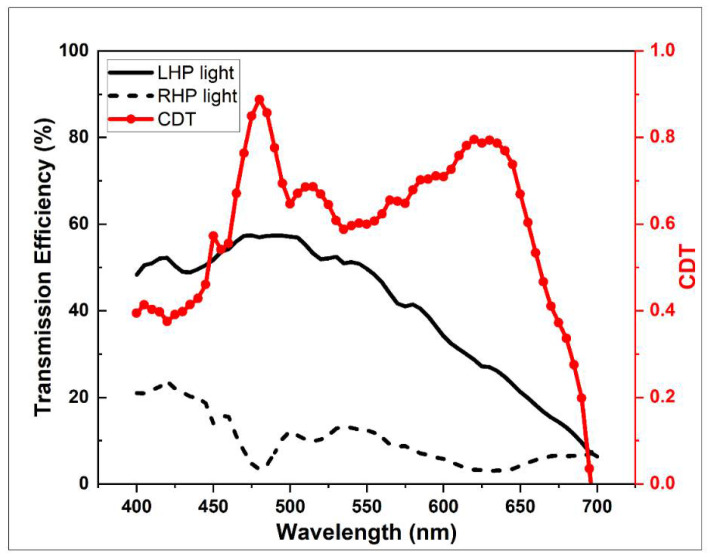
Simulation results of smaller LHDCP in a single-pixel size of 2.4 × 2.4 μm^2^: Black lines are the transmission spectra of our LHDCP under LHP and RHP incident light, the red line is the CDT calculated from the above two transmission spectra.

In order to evaluate the operation of our polarizer in a real camera system, we undertook a number of simulations in which a small array was size-matched to a commercial image sensor. Due to its underlying plasmonic nature. There is a trade-off between the optical properties (CDT and transmission efficiency) of our DCP and its size. Reducing the size of plasmonic DCP will degrade its optical properties as each block will encompass fewer nanoparticle units. Therefore, we considered a CMOS image sensor with a pixel size around 2.4 um (e.g., a Sony CMOS Exmor sensor (IMX183) with 20-M resolution). To demonstrate this issue and to obtain the transmission efficiency and CDT of our LHDCP when interfaced to this image sensor, we replaced the PBCs with PMLs and used an array of 15 × 15 elements, each 2.4 μm × 2.4 μm in size. The simulation results are shown in Figure 9. The transmission efficiency is lower than that of the previous periodic models (< 60% vs. a peak around 75%) due to the reduced number of nano-scale elements within the smaller overall area. The CDT reaches 0.5 at a lower wavelength (approximately 660 nm vs. 750 nm) for the same reason. Note that reducing the number of elements will reduce the peak CDT so it rolls off at a wavelength lower than 700 nm. This implies that 700 nm is more-or-less a hard limit for our LHDCP—it will not operate on polarized light at longer wavelengths but will simply act as a phase shifter in this region. However, its performance at visible wavelengths is still better than comparable DCP systems. Therefore, we think that our DCP is suitable for use as an image sensor with pixel sizes larger than 2.4 μm. This problem can also be addressed by trading off size against image resolution, and by arranging each DCP to cover more pixels—for example, a small block of 2 × 2 each.

Being able to determine intensity values for the RHP and LHP components means it becomes possible to calculate the fourth Stokes parameter *S*_3_, which is equivalent to the difference between the circular Jones vectors, *E,* so that $S_{3}\equiv \langle E_{r}^{2}\rangle -\langle E_{l}^{2}\rangle$, where the subscripts identify the right and left circular bases. By integrating our DCPs with linear polarizers on the image sensor, we can create a full Stokes polarization camera. Note that the linear polarizers can be achieved with Al nanogratings, which is a well-developed technology in the market. A full Stokes polarization camera with our DCPs would have a broad operating range across the visible spectrum and would be able to measure the Stokes parameters and position these on the Poincaré sphere [44], something that would be very useful in bio-inspired designs that mimic insect vision [45].

## 4. Conclusions

We have developed a dichroic circular polarizer (DCP) based on plasmonic nanostructures. The DCP comprises five layers of Al nanograting/nanocuboid surfaces embedded in SiO_2_ built onto a quartz substrate. The whole layered structure comprises only around a hundred nanometer of CMOS compatible dielectric and metal materials. Thus, this kind of DCP is not bulky and has lower optical loss that traditional DCP systems. The CDT of the device can reach more than 0.9 with maximum transmission efficiencies larger than 78% at visible wavelengths. We also demonstrate that our DCP is insensitive to angle of incidence by calculating its CDT values under varying angles up to 80°. During device integration, the operating wavelengths, transmission efficiency, and CDT can be simply tuned by varying the size, spacing, and period of the plasmonic nanostructures. Thus, the integration of these circular polarizers on a photodetector array is less complicated and much cheaper than previous methods. In addition, the CDT is still high enough to be used when the structure size shrinks to 2.4 μm, showing its capability and compatibility with most current image sensors for polarization imaging applications.

## Data Availability

The data presented in this study are available on request from the corresponding author.
